# Relation between outcomes and localisation of p-mTOR expression in gastric cancer

**DOI:** 10.1038/sj.bjc.6604915

**Published:** 2009-02-17

**Authors:** T Murayama, M Inokuchi, Y Takagi, H Yamada, K Kojima, J Kumagai, T Kawano, K Sugihara

**Affiliations:** 1 Department of Surgical Oncology, Tokyo Medical and Dental University, 1-5-45, Yushima, Bunkyo, Tokyo 113-8519, Japan; 2 Department of Translational Oncology, Tokyo Medical and Dental University, 1-5-45, Yushima, Bunkyo, Tokyo 113-8519, Japan; 3 Department of Esophagogastric Surgery, Tokyo Medical and Dental University, 1-5-45, Yushima, Bunkyo, Tokyo 113-8519, Japan; 4 Department of Human Pathology, Tokyo Medical and Dental University, 1-5-45, Yushima, Bunkyo, Tokyo 113-8519, Japan

**Keywords:** mTOR, Akt, gastric cancer

## Abstract

The mammalian target of rapamycin (mTOR), a Ser/Thr protein kinase that mediates intracellular signalling related to cell growth, proliferation, and differentiation, has received considerable interest as a possible target for cancer treatment. We evaluated the correlation of mTOR expression with clinicopathological features, outcomes, and the expression of Akt, an upstream regulator of mTOR, in gastric cancer. Tumour samples were obtained from 109 patients with gastric adenocarcinomas who underwent a radical gastrectomy. The expressions of phosphorylated mTOR (p-mTOR) and phosphorylated Akt (p-Akt) in the cytoplasm and in the nucleus were analysed by immunohistochemical staining. Cytoplasmic p-mTOR expression positively correlated with the depth of tumour invasion (T1 *vs* T2–4, *P*=0.003), involved lymph nodes (*P*=0.010), and tumour stage (I *vs* II–IV, *P*=0.002). In contrast, nuclear p-mTOR expression negatively correlated with these variables (*P*<0.001,=0.035, and <0.001). Cytoplasmic p-mTOR expression was associated with significantly poorer relapse-free survival (RFS, *P*=0.037) and overall survival (OS, *P*=0.024), whereas nuclear p-mTOR expression was associated with better RFS and OS (*P*=0.029, 0.059). Neither cytoplasmic nor nuclear p-Akt expression was associated with any clinicopathological factor or with survival. Localisation of p-mTOR may play an important role in tumour progression and outcomes in patients with gastric cancer.

Gastric cancer is one of the most common malignancies worldwide and ranks as the second leading cause of cancer-related death (Kamangar *et al*, 2006). Outcomes remain poor in patients with unresectable or metastatic gastric cancer. Their median survival has improved, but is still only about 1 year, even with intensive chemotherapy (Van Cutsem *et al*, 2006; Cunningham *et al*, 2008; Koizumi *et al*, 2008). Anticancer drugs aimed at molecular regulators, including epidermal growth factor receptor (EGFR or HER1), its homologue c-erb-2 (HER2), have been developed and shown to be effective in the breast, lung, and colon cancers (Slamon *et al*, 2001; Thatcher *et al*, 2005; Gatzemeier *et al*, 2007; Sobrero *et al*, 2008). These drugs have been evaluated in advanced gastric cancer, but two EGFR tyrosine kinase inhibitors were found to be ineffective (Dragovich *et al*, 2006; Rojo *et al*, 2006).

The mammalian target of rapamycin (mTOR) has received considerable attention as a possible target for cancer treatment (Huang and Houghton, 2002). mTOR is a Ser/Thr protein kinase that mediates nutrient-dependent intracellular signalling related to cell growth, proliferation, and differentiation. It also functions by integrating extracellular signals such as growth factors. mTOR promotes translation initiation by phosphorylating two targets, ribosomal p70S6 kinase (S6K1) and eukaryotic translation initiation factor 4E binding protein 1 (4E-BP1) (Schmelzle and Hall, 2000; Petroulakis *et al*, 2006). Rapamycin is a macrolide antibiotic and an immunosuppressive agent that inhibits mTOR. Its antiproliferative effect is mediated through the formation of an active complex. Rapamycin also suppresses angiogenesis by decreasing the production of vascular endothelial growth factor (Guba *et al*, 2002). mTOR inhibitor has shown promising efficacy in a phase III clinical trial in patients with metastatic renal cell cancer (Motzer *et al*, 2008). The anticancer effect of RAD001, a rapamycin analogue, has also been shown in advanced gastric cancer (Muro *et al*, 2008). However, few studies have assessed correlations of mTOR expression in human cancers with either clinocopathological features or outcomes (Zhou *et al*, 2004; Rajan *et al*, 2008).

Akt, also known as protein kinase B, is an upstream regulator of mTOR. Three isoforms of Akt have been identified, Akt1, Akt2, and Akt3. Increased expression of Akt isoform was reported in various cancers. Akt is a key intermediate of signalling pathways that regulate cellular processes involved in cell growth, proliferation, survival, and neo-vascularization. Akt has also been shown to play an important role in the chemotherapeutic resistance of tumour cells. The Akt signal transduction pathway is thus considered a promising target for chemotherapy (Altomare and Testa, 2005). However, elevated Akt activity was not associated with tumour progression or poor outcomes in several studies (Tsao *et al*, 2003; Shah *et al*, 2005; Chadha *et al*, 2006). Phosphorylated Akt (p-Akt) expression was reported in preneoplastic lesions such as bronchial dysplasia, suggesting that activated Akt has an early role in tumour progression (Tsao *et al*, 2003; Balsara *et al*, 2004). Activated Akt has also been frequently observed in gastric cancer (Bellacosa *et al*, 2005), although two studies showed no correlation of activated Akt with tumour progression or poor survival (Nam *et al*, 2003; Oki *et al*, 2005).

Both Akt and mTOR are activated by phosphorylation. This study examined correlations of phosphorylated mTOR (p-mTOR) expression with clinicopathological features, outcomes, and p-Akt expression in gastric cancer. The expressions of p-mTOR and p-Akt were evaluated immunohistochemically.

## Patients and methods

### Patients

The study group comprised 109 patients with primary gastric adenocarcinomas who underwent curative gastrectomy (R0) from January 1999 to December 2002 at the Department of Esophagogastric Surgery, Tokyo Medical and Dental University. Each tumour was classified according to the tumour-node-metastasis classification recommended by the International Union against Cancer (UICC). All patients were evaluated for recurrent disease by examination of tumour markers or by diagnostic imaging, including computed tomography, ultrasonography, magnetic resonance imaging, and endoscopy, every 3–6 months. No patient received neoadjuvant therapy, although five patients with stage IV disease received postoperative adjuvant chemotherapy with S-1.

The median follow-up time was 1953 days (range: 50–3197). Recurrent disease was diagnosed in 29 patients (27%) and was the cause of death in 28 of these patients.

### Immunostaining of p-mTOR and p-Akt

Immunohistochemical staining was carried out by the streptavidin–biotin method using a Histofine SAB-PO kit (Nichirei Co., Tokyo, Japan). Polyclonal rabbit anti-human antibodies against p-mTOR (Ser^2448^) and p-Akt (Ser^473^) were purchased from Cell Signaling Technology, Inc. (Beverly, MA, USA). All available haematoxylin and eosin stained slides of the surgical specimens were reviewed. For each case, representative paraffin blocks were selected for immunohistochemical studies. Three-micrometer-thick sections were cut from formalin-fixed, paraffin-embedded tissue blocks. After deparaffinisation and rehydration, antigen retrieval treatment was carried out at 121°C (autoclave) for 5 min in 10 nmol l^−1^ sodium citrate buffer (pH9.0), followed by treatment with 3% hydrogen peroxide for 15 min to quench endogenous peroxidase activity. Nonspecific binding was blocked by treating the slides with 5% EzBlock (including 5% normal goat serum and 0.1% Tween 20) for 60 min at room temperature. The slides were incubated with primary antibodies including p-mTOR (dilution 1 : 50) and p-Akt (1 : 50) overnight at 4°C. Immunodetection was performed by the conventional streptavidin–biotin method with a Nichirei SAB-PO kit. The slides were counterstained with 1% Mayer's haematoxylin.

The p-mTOR and p-Akt levels were classified into three groups based on both staining intensity and positive frequency according to the scoring method described by (Zhou *et al*, 2004). Tumours in which <10% of cells were weakly stained were scored as 0, tumours in which >10% of cells were weakly stained or <20% of cells were strongly stained were scored as 1, and tumours in which >20% of cells were strongly stained were scored as 2. A score of ‘1’ or ‘2’ was defined as ‘positive’ expression, and a score of ‘0’ was defined as ‘negative’ expression. We counted stained cells under a microscope to derive the scores. Cytoplasmic staining and nuclear staining were evaluated separately.

### Statistical analysis

The *χ*^2^ test was used to test possible associations between the expression of p-mTOR or p-Akt and clinicopathological factors. It was also used to assess correlations between p-mTOR and p-Akt expressions. The Mann–Whitney *U*-test was used to analyse the relation between each type of expression and age. Kaplan–Meier curves were plotted to assess the effects of p-mTOR and p-Akt expressions on relapse-free survival (RFS) and overall survival (OS). Survival curves were compared using the log-rank test. *P*-values of 0.05 or less were considered to indicate statistical significance. Multivariate proportional Cox models were used to assess the prognostic significance of p-mTOR and p-Akt expressions and of several clinicopathological factors. Statistical analysis was carried out with the use of SPSS Base, version 11.0 and SPSS Advanced Models, version 11.0 (SPSS Inc, Chicago, IL, USA) software.

## Results

**Figure 1:**
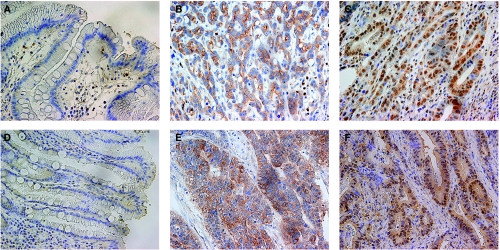
No expression of p-mTOR (**A**) or p-Akt (**D**) was detected in normal gastric mucosa. Representative gastric carcinomas showing immunostaining for p-mTOR predominantly in the cytoplasm (**B**) and predominantly in the nucleus (**C**); immunostaining for p-Akt predominantly in the cytoplasm (**E**) and predominantly in the nucleus (**F**), magnification; × 400.

Cytoplasmic expression of p-mTOR was found in 69 (63%) of all tumours, and nuclear expression was found in 33 (30%). Cytoplasmic expression of p-Akt was observed in 94 (86%) tumours, and nuclear expression was observed in 45 (43%). No p-mTOR or p-Akt staining was detected in normal gastric mucosa (Figure 1).

There was no correlation between p-mTOR and p-Akt expression, but many tumours with cytoplasmic p-mTOR expression showed cytoplasmic p-Akt expression. Nearly all the tumours with nuclear p-mTOR or p-Akt expression showed cytoplasmic expression of the same molecule (Table 1).

The cytoplasmic expression of p-mTOR positively correlated with the depth of tumour invasion (T1 *vs* T2–4; *P*=0.003), lymph node involvement (*P*=0.010), and UICC stage (I *vs* II–IV; *P*=0.002). Nuclear p-mTOR expression negatively correlated with the depth of tumour invasion, lymph node involvement, and the UICC stage (*P*<0.001,=0.035 and <0.001, respectively). Neither cytoplasmic nor nuclear p-Akt expression was related to any clinicopathological factor. The rates of cytoplasmic p-mTOR and p-Akt expressions were slightly, but not significantly, higher among younger patients (Tables 2 and 3).

**Figure 2:**
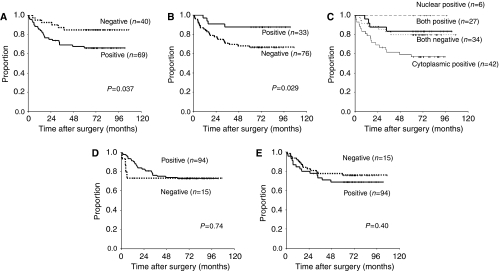
Kaplan–Meier curves for the relapse-free survival of patients with expression of cytoplasmic p-mTOR (**A**), nuclear p-mTOR (**B**), cytoplasmic p-Akt (**D**), and both nuclear p-Akt and cytoplasmic p-Akt (**E**). Kaplan–Meier curves for the four subgroups classified according to p-mTOR expression are shown in (**C**).

Patients with cytoplasmic p-mTOR expression had significantly shorter RFS and OS than those without cytoplasmic p-mTOR expression (*P*=0.037, 0.024, respectively). In contrast, nuclear p-mTOR expression was associated with significantly longer RFS (*P*=0.029), as well as with slightly, but not significantly, longer OS (*P*=0.053). We next classified patients into the following four subgroups according to the p-mTOR expression of their tumours and analysed survival: patients with positive expressions of both cytoplasmic and nuclear p-mTOR (group A), positive expression of only cytoplasmic p-mTOR (B), positive expression of only nuclear p-mTOR (C), and negative expressions of both cytoplasmic and nuclear p-mTOR (D). Survival was significantly poorer in group B than in the other three groups (*vs* group A, C, and D; *P*=0.031, 0.049, and 0.036, respectively). There were no other significant differences in this analysis. In contrast, neither cytoplasmic nor nuclear expression of p-Akt was associated with either RFS (*P*=0.74, 0.40, respectively) or OS (*P*=0.77, 0.65, respectively) (Figure 2).

The prognostic relevance of p-mTOR and p-Akt expression was assessed using a multivariate proportional-hazards model adjusted for established clinical prognostic factors (i.e., depth of tumour invasion, lymph node involvement, histological type, sex, and age) (Table 4). The depth of tumour invasion and lymph node involvement were independent prognostic factors (hazard ratio (HR)=6.62, 95% confidence interval (CI) 1.32–33.4, *P*=0.022; HR=3.08, 95% CI 1.04–9.21, *P*=0.043, respectively), but cytoplasmic expression of p-mTOR was not independent (HR=1.42, 95% CI 0.51–3.97, *P*=0.51).

## Discussion

Our study showed that p-mTOR expression was significantly related to tumour progression and outcomes in patients with gastric cancer. Interestingly, the cytoplasmic expression of p-mTOR positively correlated with factors related to tumour progression and poor outcomes in gastric cancer, whereas the nuclear expression of p-mTOR negatively correlated with such factors. This finding suggests that changes in the localisation of p-mTOR may be involved in tumour progression. Other investigators reported that m-TOR is mainly localised in the cytoplasm (Janus *et al*, 2005; Bachmann *et al*, 2006), although a small fraction of mTOR is found at a steady state in the nucleus in both normal and malignant cells (Zhang *et al*, 2002). mTOR is part of two distinct complexes: mTORC1 containing raptor and a mammalian orthologue of yeast Lst8p (mLST8; also known as G*β*L), and mTORC2 containing rictor, mLST8, and sin1 (also known as mitogen-activated protein-kinase-associated protein 1) (Sarbassov *et al*, 2005; Petroulakis *et al*, 2006; Rosner and Hengstschläger, 2008). mTORC1 was predominantly found in the cytoplasm of fibroblast cells (Rosner and Hengstschläger, 2008), although its distribution was not clear in cancer cells. mTORC1 pathway promotes cell growth and proliferation by activating mRNA translation and ribosome biogenesis and by inhibiting autophagy through activation of S6K1 and inhibition of 4E-BP1. S6K1 drives translation of 5′TOP (terminal oligopyrimidine tract) mRNAs, and 4E-BP1 inhibits the mRNA cap-binding protein elF4E (Schmelzle and Hall, 2000). 4E-BP1 and S6K1 were detected exclusively in the cytoplasm of cancer cells (Zhang *et al*, 2002). We suggest that increased cytoplasmic mTORC1 complex may activate signalling to cytoplasmic S6K1 and 4E-BP1 and contribute to tumour progression, although 4E-BP1 and S6K1 were not investigated in this study. In addition, mTOR shuttles between the nucleus and the cytoplasm (Zhang *et al*, 2002; Bachmann *et al*, 2006). The nuclear import of mTOR has an important role in activating its cytoplasmic signalling (Bachmann *et al*, 2006). However, the mechanism of nuclear transportation of mTOR and the function of nuclear mTOR remain unclear. Only substrates activated by nuclear p-mTOR may not be adequate to promote tumour growth because nuclear p-mTOR was found more frequently in early-stage disease. On the other hand, mTORC2 is required for Akt phosphorylation on Ser473 to achieve full activation (Sarbassov *et al*, 2005). Activated Akt was not related to either tumour progression or outcomes in this study, therefore, mTORC2 also may not be related to these variables. Rosner and Hengstschläger provided evidence that cytoplasmic raptor had a higher affinity for mTOR than nuclear raptor (Rosner and Hengstschläger, 2008). They proposed distinct mechanisms for regulation of mTOR in the cytoplasm and the nucleus, and such mechanisms may be associated with clinical outcomes.

In this study, 31% of tumours showed no activation of p-mTOR, despite of a high frequency of activated Akt. The rate of negative p-mTOR in gastric cancer was similar to that in another study (Lang *et al*, 2007). That study also showed that negative expression of p-mTOR was observed in normal gastric mucosa, whereas its positive expression was more frequent in advanced disease. In tumours without p-mTOR expression, another signalling pathway, such as Erk, may be activated. In pancreatic cancer, high expression of phosphorylated Erk was associated with shorter survival, whereas high expression of p-Akt was associated with longer survival (Chadha *et al*, 2006). In breast cancer, p-mTOR expression is predominantly detected in the cytoplasm and is significantly associated with short RFS, but does not correlate with any clinicopathological factors, including stage, histological grade, and lymph node involvement (Zhou *et al*, 2004). Rajan *et al* found that p-mTOR expression is unrelated to survival in pancreatic cancer (Rajan *et al*, 2008). Boone *et al* reported that p-mTOR expression is only associated with a lesser degree of tumour differentiation (Boone *et al*, 2008). To our knowledge, however, no earlier study has assessed nuclear p-mTOR expression. Our study is the first to show that different localisation of mTOR expression (i.e., cytoplasmic *vs* nuclear) was associated with different outcomes in patients with cancer.

Phosphorylated Akt activates many downstream targets, including mTOR, and is thought to play a role in tumour progression. However, earlier studies of Akt expression in human cancers have yielded conflicting results. In pancreatic cancer, one study showed a correlation between higher p-Akt expression and shorter survival (Yamamoto *et al*, 2004), whereas another study showed the opposite (Chadha *et al*, 2006). Higher p-Akt expression was associated with poor outcomes in breast cancer (Zhou *et al*, 2004), and with lymph node metastasis or advanced disease stage in colorectal cancer (Itoh *et al*, 2002). On the other hand, increased p-Akt expression correlated with favourable outcomes in non-small-cell lung cancer (Shah *et al*, 2005). In gastric cancer, Nam *et al* reported that tumours with p-Akt expression are associated with the absence of lymph node metastasis and with longer survival in early-stage disease (Nam *et al*, 2003). In our study, p-Akt expression was frequently observed in both early and advanced gastric cancers and was not related to clinicopathological factors or survival. These discrepancies may be partly explained by differences in the type of cancer studied, in the system used to classify p-Akt staining, or in the expression of downstream targets of p-AKt, such as mTOR.

Several inhibitors of mTOR kinase have been evaluated in various solid tumours, including renal, breast, pancreatic, and endometrial cancer, and their anticancer efficacy has been shown (Janus *et al*, 2005). The mTORC1 complex is sensitive to rapamycin (Janus *et al*, 2005; Sarbassov *et al*, 2005). As mTORC1 complex is mainly found in the cytoplasm, tumours with cytoplasmic expression of mTOR may be more sensitive to mTOR inhibitors than those with nuclear expression of mTOR. In addition, Akt requires mTORC2 to achieve its full activation, suggesting that inhibition of mTORC2 is essential for preventing the progression of tumours with highly activated Akt/mTOR signalling.

In conclusion, cytoplasmic p-mTOR expression was associated with tumour progression and poor survival in gastric cancer; the opposite results were obtained for nuclear p-mTOR expression. Localisation of p-mTOR might thus be critical to tumour progression and outcomes in patients with gastric cancer.

## Figures and Tables

**Table 1:** Correlations among the cytoplasmic and nuclear expressions of p-mTOR and p-Akt

	**p-mTOR (nucleus)**			**p-Akt (cytoplasmic)**			**p-Akt (nucleus)**		
	**Negative**	**Positive**	***P-*value**	**Negative**	**Positive**	***P-*value**	**Negative**	**Positive**	***P-*value**
*p-mTOR (cytoplasmic)*									
Negative	34	6	0.015	9	31	0.084	27	13	0.16
Positive	42	27		6	63		37	32	
									
*p-mTOR (nucleus)*									
Negative				10	66	0.77	47	29	0.31
Positive				5	28		17	16	
									
*p-Akt (cytoplasmic)*									
Negative							15	0	<0.001
Positive							49	45	

Abbreviations: p-Akt=phosphorylated Akt; p-mTOR=phosphorylated mTOR.

**Table 2:** Correlations between p-mTOR expression and clinicopathological factors

	**All**	**Cytoplasmic p-mTOR**			**Nuclear p-mTOR**		
	***n* (%)**	**Negative**	**Positive**	***P***-**value**	**Negative**	**Positive**	***P***-**value**
*Sex*							
Male	77 (71)	26	51	0.33	54	23	>0.99
Female	32 (29)	14	18		22	10	
Median age (range)	65 (35–85)	62 (35–79)	66 (43–85)	0.079	65 (35–85)	64 (51–81)	0.77
							
*Depth of invasion*							
T1	45 (41)	24	21	0.003	23	22	<0.001
T2/3/4	41 (38)/20 (18)/4 (4)	16	48		53	11	
*LN metastasis*							
Positive (N1/2/3)	33 (30)/17 (16)/3 (3)	13	40	0.010	42	11	0.035
Negative (N0)	56 (51)	27	29		34	22	
							
*Stage*							
I	59 (54)	29	29	0.002	31	27	<0.001
II/III/IV	26 (24)/16 (15)/8 (7)	11	40		45	6	
							
*Recurrence*							
Positive	29 (27)	6	23	0.062	25	4	0.033
Negative	80 (73)	34	46		51	29	
							
*Histopathology*							
Intestinal	40 (37)	13	27	0.49	25	15	0.21
Diffuse	69 (63)	27	42		51	18	

Abbreviations: LN metastasis=lymph node metastasis; p-mTOR=phosphorylated mTOR.

**Table 3:** Correlations between p-Akt expression and clinicopathological factors

	**All**	**Cytoplasmic p-Akt**			**Nuclear p-Akt**		
	***n* (%)**	**Negative**	**Positive**	***P-*value**	**Negative**	**Positive**	***P-*value**
*Sex*							
Male	77 (71)	9	68	0.50	45	32	0.93
Female	32 (29)	6	26		19	13	
Median age (range)	65 (35–85)	58 (35–72)	65 (43–85)	0.060	64 (35–82)	66 (43–85)	0.81
							
*Depth of invasion*							
T1	45 (41)	7	38	0.86	26	19	0.87
T2/3/4	41 (38)/20 (18)/4(4)	8	56		38	26	
							
*LN metastasis*							
Positive (N1/2/3)	33 (30)/17(16)/3(3)	6	47	0.66	30	23	0.66
Negative (N0)	56 (51)	9	47		34	22	
							
*Stage*							
I	59 (54)	10	48	0.28	37	21	0.25
II/III/IV	26 (24)/16 (15)/8 (7)	5	46		27	24	
							
*Recurrence*							
Positive	29 (27)	4	25	>0.99	15	14	0.37
Negative	80 (73)	11	69		49	31	
							
*Histopathology*							
Intestinal	40 (37)	4	36	0.57	24	16	0.84
Diffuse	69 (63)	11	58		40	29	

Abbreviations: LN metastasis=lymph node metastasis; p-Akt=phosphorylated Akt.

**Table 4:** Prognostic factors in a multivariate Cox proportional-hazards regression model

	**HR**	**95%Cl**	***P*-value**
Age	0.98	0.94–10.2	0.35
Sex; female *vs* male	1.23	0.49–3.11	0.66
Pathological type; intestinal *vs* diffuse	0.88	0.38–2.05	0.77
Depth of invasion; T1 *vs* T2–4	6.62	1.32–33.4	0.022
Involved lymph nodes	3.09	1.04–9.21	0.043
Cytoplasmic p-mTOR	1.42	0.51–3.97	0.51
Nuclear p-mTOR	0.57	0.18–1.77	0.33
Cytoplasmic p-Akt	0.35	0.11–1.16	0.086
Nuclear p-Akt	0.16	0.80–3.99	0.16

Abbreviations: CI=confidence interval; HR=hazard ratio; p-Akt=phosphorylated Akt; p-mTOR=phosphorylated mTOR.
